# Crosstalk between protein kinases Pho85-Pho80 and Rim11 regulates Pah1 phosphatidate phosphatase function in lipid synthesis

**DOI:** 10.1016/j.jbc.2026.113475

**Published:** 2026-08-24

**Authors:** Shoily Khondker, Emily R. Bostrom, Taylor Carmon, Kam Shan Hu, Andrew Privalov, Gil-Soo Han, George M. Carman

**Affiliations:** Department of Food Science and the Rutgers Center for Lipid Research, Rutgers University, New Brunswick, New Jersey, USA

**Keywords:** triacylglycerol, phospholipid, phosphatidate, diacylglycerol, Pah1 phosphatidate phosphatase, protein phosphatase Nem1-Spo7, protein kinase Pho85-Pho80, protein kinase Rim11, lipid synthesis regulation

## Abstract

In *Saccharomyces cerevisiae*, the Pah1 phosphatidate (PA) phosphatase is essential for the synthesis of the storage lipid triacylglycerol. Through its utilization of the substrate PA, Pah1 also exerts a negative regulatory effect on phospholipid synthesis, a process required for cell growth and proliferation. The function of Pah1 is controlled by its membrane association, which is regulated by phosphorylation and dephosphorylation. Multiple protein kinases phosphorylate Pah1 to maintain it in the cytosol, whereas recruitment and dephosphorylation by the Nem1-Spo7 phosphatase complex promotes its translocation to the nuclear/endoplasmic reticulum membrane. Cyclin-dependent kinase Pho85-Pho80 and glycogen synthase kinase Rim11 both phosphorylate Pah1, thereby influencing its localization and inhibiting its PA phosphatase activity. In this study, we demonstrate that Rim11 is a *bona fide* substrate of the Pho85-Pho80 complex, with kinase activity dependent on Rim11, ATP, and reaction time. Pho85-Pho80 phosphorylates Rim11 at Ser-8, which enhances the ability of Rim11 to phosphorylate Pah1. A Rim11 peptide containing the Ser-8 site served as a substrate for Pho85-Pho80 and inhibited the phosphorylation of full-length Rim11 by the protein kinase complex. Mutational analysis revealed that the phosphorylation state of Pah1, as mediated by Pho85-Pho80 and Rim11, regulates the expression of the key phospholipid-synthesizing enzyme Cho1 phosphatidylserine synthase. Collectively, these findings deepen our understanding of how Pho85-Pho80 modulates Rim11 through phosphorylation and highlight the interconnected roles of these protein kinases in controlling Pah1 function in lipid synthesis.

In the yeast *Saccharomyces cerevisiae*, Pah1 phosphatidate (PA) phosphatase has emerged as a key regulatory enzyme in lipid synthesis (1, 2, 3, 4, 5, 6). By catalyzing the dephosphorylation of PA to produce diacylglycerol (DAG) (7), the enzyme reciprocally controls the levels of two key intermediates required for the synthesis of membrane phospholipids, which are essential for cell growth, and for the synthesis of triacylglycerol (TAG), the storage lipid found in lipid droplets (3, 5, 6, 8) (Fig. 1*A*). PA and DAG also have impacts on cellular processes that include membrane fission and fusion (9, 10, 11, 12, 13, 14), vesicular trafficking (15, 16, 17, 18, 19), lipid signaling (6, 20, 21, 22, 23, 24, 25), and in particular, PA regulates phospholipid synthesis gene expression *via* the Henry (Ino2-Ino4/Opi1) regulatory circuit (26, 27, 28) (Fig. 1*A*). The importance of Pah1 in maintaining the PA/DAG balance is exemplified by deleterious phenotypes observed in the *pah1*Δ mutant (6). These include abnormal nuclear membrane structure, impaired lipid droplet formation, fatty acid-induced lipotoxicity, defects in vacuole fusion and autophagy, apoptosis, and a reduced chronological lifespan (7, 27, 29, 30, 31, 32, 33, 34, 35, 36, 37, 38, 39, 40, 41, 42).

**Figure 1:**
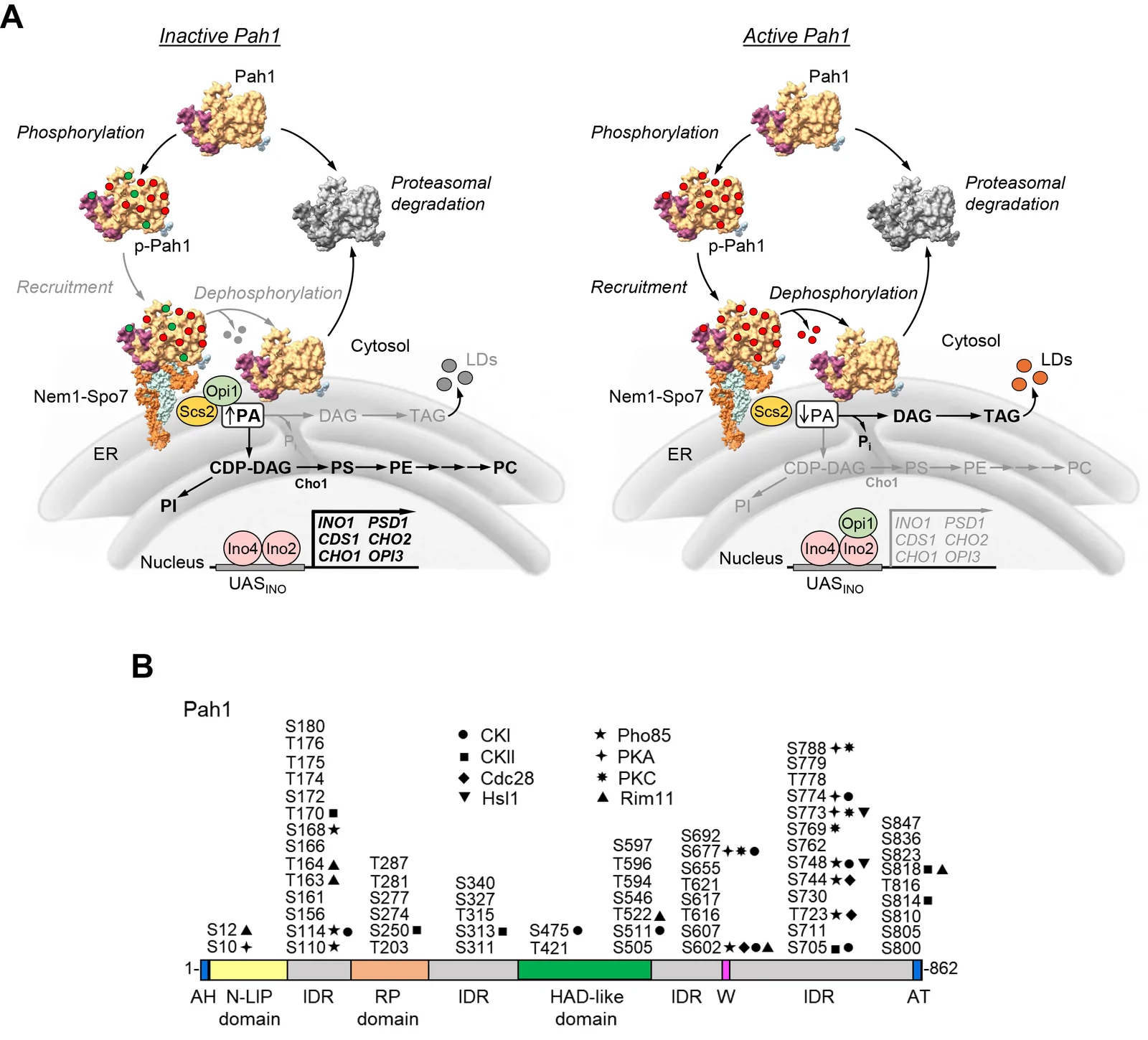
**Model for the translocation of Pah1 for its function in TAG synthesis and in the transcriptional regulation of phospholipid synthesis genes, and the phosphorylation sites in Pah1.***A*, the figure shows simplified AlphaFold3 (128) structures of Pah1 (N-LIP, *purple*; HAD-like, *gold*) and the Nem1 (*orange*)-Spo7 (*light blue*) complex, the bifurcation of PA for the synthesis of TAG and phospholipids, and the Henry regulatory circuit involving the Ino2-Ino4 activator complex, the Opi1 repressor, and the UAS_INO_ element of phospholipid synthesis genes (3, 6, 8, 26). For simplicity, only a subset of all UAS_INO_-containing genes are represented in the figure. Pah1 in the cytosol is phosphorylated (p-Pah1) by multiple protein kinases for protection against proteasomal degradation (*gray shading*). The p-Pah1 is stable, but functionally inactive due to its cytosolic sequestration apart from the nuclear/ER membrane-associated substrate PA. The cytosolic p-Pah1 is recruited and dephosphorylated by the nuclear/ER-associated Nem1-Spo7 complex, and the resulting dephosphorylated Pah1 associates with the membrane for its activity on PA to produce DAG that is converted to TAG stored in lipid droplets. Following rounds of catalysis, Pah1 dissociating from the nuclear/ER membrane is subject to proteasomal degradation. *Left*, an alternative phosphorylation state (indicated by *red* and *green dots*) of p-Pah1 reduces its recruitment and dephosphorylation. The attenuation of Pah1 function reduces TAG synthesis and lipid droplet formation (*gray arrows* and *letters*). When Pah1 is less active, the elevated level of PA increases the tethering of Opi1 to the nuclear/ER membrane *via* its interaction with PA and Scs2 (3, 6, 8, 26), which enables the transcriptional activation (*bold arrow* and *letters*) of the UAS_INO_-containing genes by the Ino2-Ino4 complex. The induced expression of enzymes in the CDP-DAG pathway increases the synthesis of membrane phospholipids (*bold arrows* and *letters*) (3, 6, 8, 26). Cho1 phosphatidylserine synthase catalyzes the committed step for the synthesis of phosphatidylcholine *via* the CDP-DAG pathway. *Right*, when Pah1 is active, the reduced level of PA facilitates the production of free Opi1, which is not associated with PA and the membrane protein Scs2, and its nuclear entry to repress the transcription of the UAS_INO_-containing genes (*thin gray arrow and letters*) *via* interaction with the Ino2 subunit of the Ino2-Ino4 complex. The repression of the CDP-DAG pathway genes results in the reduction of phospholipid synthesis (*gray arrows* and *letters*) and a concomitant increase in the synthesis of TAG and lipid droplets (*bold arrows* and *letters*). *B*, the N-LIP and the haloacid dehalogenase (HAD)-like domains of Pah1 are required for PA phosphatase activity (7, 129). The N-terminal amphipathic helix (*AH*) is responsible for interaction with the membrane (43). The regulation of phosphorylation (RP) domain facilitates Pah1 phosphorylation (130). The essential tryptophan contained within the WRDPLVDID domain is required for the *in vivo* catalytic function (87, 131). The acidic tail (*AT*) associates with the Nem1-Spo7 complex (69) through the ionic interaction with the C-terminal basic tail of Spo7 (74). The intrinsically disordered regions (IDRs) contain most of the phosphorylation sites that serve for interaction with the Nem1-Spo7 complex. The serine (*S*) and threonine (*T*) residues known to be phosphorylated (32, 47, 48, 49, 50, 51, 52, 53, 54, 55, 56, 57, 58) are grouped at their approximate regions in the Pah1 protein. The sites phosphorylated by casein kinase I (*CKI*) (63), casein kinase II (*CKII*) (64), Cdc28 (44), Hsl1 (65), Pho85 (45), protein kinase A (*PKA*) (61), protein kinase C (*PKC*) (62), and Rim11 (66) are indicated. CDP-DAG, CDP-diacylglycerol; DAG, diacylglycerol; LDs, lipid droplets; PA, phosphatidate; PC, phosphatidylcholine; PI, phosphatidylinositol; PS, phosphatidylserine; TAG, triacylglycerol.

The cellular function of Pah1 depends on its membrane localization that is controlled by the posttranslational modifications of phosphorylation and dephosphorylation (32, 43, 44, 45, 46) (Fig. 1*A*). Pah1 is one of the most heavily phosphorylated proteins in *S. cerevisiae* with 66 serine/threonine residues (32, 47, 48, 49, 50, 51, 52, 53, 54, 55, 56, 57, 58) being the target sites of multiple protein kinases (59) (Fig. 1*B*). Many sites of Pah1 phosphorylation are controlled by growth phase and/or environmental and chemical perturbations (60). The protein kinase-phosphosite relationship has been confirmed for cyclin-dependent protein kinases Pho85 (45) and Cdc28 (44), protein kinases A (61) and C (62), casein kinases I (63) and II (64), septin-associated protein kinase Hsl1 (65), and glycogen synthase kinase Rim11 (66) (Fig. 1*B*). The effects of phosphorylation on Pah1 are multifaceted affecting its location, activity, and stability (46). In general, phosphorylation suppresses Pah1 function by promoting its retention in the cytosol, thereby separating the enzyme from its membrane-associated substrate PA (Fig. 1*A*). The impact of phosphorylation on activity depends on the protein kinase involved. For example, phosphorylation by Pho85–Pho80 (45), Rim11 (66), and Hsl1 (65) decreases activity, whereas phosphorylation by casein kinase I (63) and protein kinase C (62) enhances activity. Phosphorylation generally increases Pah1 stability and abundance (67, 68). However, phosphorylation by protein kinase C has the opposite effect, promoting Pah1 destabilization and degradation, particularly when Pah1 has not been previously phosphorylated by Pho85–Pho80 (62).

The Nem1 (catalytic subunit)-Spo7 (regulatory subunit) phosphatase complex, which resides at the nuclear/ER membrane, is the essential regulator of Pah1 for its catalytic function on the membrane (Fig. 1*A*). The phosphatase complex recruits and dephosphorylates Pah1 so that it can associate with the membrane, recognize PA, and catalyze the dephosphorylation reaction (29, 32, 43, 69, 70, 71) (Fig. 1*A*). The localization of Pah1 to the nuclear/ER membrane (72, 73) is accomplished by both subunits of the phosphatase complex: Nem1 recognizes and binds to phosphorylated Pah1 as its substrate (32, 70), and the basic tail of Spo7 interacts with the acidic tail of Pah1 (69, 74). The dephosphorylation of Pah1 affects its catalytic function and protein stability (Fig. 1*A*). While dephosphorylated Pah1 is more active (70), thereby promoting TAG synthesis, it is vulnerable to proteasomal degradation (68), providing a mechanism for activity control and enzyme turnover (73).

Of the protein kinases that phosphorylate Pah1, Pho85-Pho80 exerts a major effect on its localization, catalytic activity, and protein stability (45, 46, 68, 70). Pho85-Pho80 phosphorylates Pah1 on seven sites: Ser-110, Ser-114, Ser-168, Ser-602, Thr-723, Ser-744, and Ser-748 (45) (Fig. 1*B*). Mutant Pah1 (7A) deficient in phosphorylation on these residues bypasses the requirement of the Nem1-Spo7 complex for its recruitment, dephosphorylation, and activation at the nuclear/ER membrane (32, 44, 45, 70, 75). Thus, the Pho85-Pho80 phosphorylation of Pah1 ensures its cellular function through the Nem1-Spo7 complex-mediated translocation. The Pah1 phosphorylation also creates a hierarchical effect on its subsequent phosphorylation by other protein kinases (63, 66). For instance, the Pho85-Pho80 prephosphorylation of Pah1 enhances its phosphorylation by Rim11 (66), but reduces its phosphorylation by casein kinase I (63).

Rim11 phosphorylates Pah1 on multiple serine (Ser-12, Ser-602, Ser-818) and threonine (Thr-163, Thr-164, and Thr-522) residues (66) (Fig. 1*B*). Mutational analysis of the major phosphorylation sites (Thr-163, Thr-164, and Ser-602) indicates that like Pho85-Pho80 (45), Rim11 ensures the Nem1-Spo7 complex-dependent localization of Pah1 to the nuclear/ER membrane (66). Rim11-phosphorylated Pah1 is a substrate for dephosphorylation by Nem1-Spo7 (66). Rim11 can phosphorylate Pah1 without its prior phosphorylation (66), however, the kinase activity increases when the substrate is phosphorylated by Pho85-Pho80, even though Ser-602 is a shared phosphosite for both protein kinases (45, 66) (Fig. 1*B*). The stimulatory effect of Pho85-Pho80 may be due to the priming phosphorylation of Pah1 on Ser-168 by Pho85-Pho80 (66). Conversely, the prior phosphorylation of Pah1 by Rim11 has little effect on its subsequent phosphorylation by Pho85-Pho80 (66). In this case, it is likely that Pho85-Pho80 phosphorylates Pah1 on its six additional phosphosites besides Ser-602 (45).

Rim11 has been identified by phosphoproteomic studies to contain eight phosphorylation sites: seven serine/threonine residues (Ser-5, Ser-8, Ser-12, Ser-17, Thr-193, Ser-198, and Ser-202) and one tyrosine residue (Tyr-199) (47, 49, 50, 52, 53, 55, 56, 58, 76, 77, 78). Except for the phosphorylation of Tyr-199, which is essential for catalytic activity and occurs by autophosphorylation (79, 80), protein kinases phosphorylating the other phosphosites are unknown. Motif analysis of the phosphosites indicates that Ser-8 lies within the minimal (Ser/Thr)-Pro motif as a putative target for Pho85-Pho80 (81). In this study, we demonstrate that Rim11 is a *bona fide* substrate of Pho85-Pho80, and its phosphorylation sites are Ser-8 (major) and the previously unrecognized residue Thr-258 (minor). In addition, we show that the Pho85-Pho80 protein kinase-mediated phosphorylation of Rim11 on Ser-8 stimulates its ability to phosphorylate Pah1. Through a mutagenesis analysis, we also show that the phosphorylation state of Pah1, as mediated by Pho85-Pho80 and Rim11, regulates the expression of the key phospholipid synthesis enzyme Cho1 phosphatidylserine (PS) synthase. Collectively, these results enhance our understanding of how the Pho85-Pho80 complex regulates Rim11 *via* phosphorylation and highlight the interconnected roles of the protein kinases in controlling the Pah1 function in lipid synthesis.

## Results

### Rim11 is a *bona fide* substrate of Pho85-Pho80

In this work, we examined the hypothesis that the Pho85-Pho80 complex phosphorylates Rim11, and that this modification facilitates the ability of Rim11 to phosphorylate Pah1. Our experiments utilized purified preparations of the epitope-tagged Pho85-Pho80, Rim11 (WT and S8A mutant), and Pah1 (Fig. 2). Whereas the active and unphosphorylated forms of Pho85-Pho80 (81) and Pah1 (7) were heterologously expressed and purified from *Escherichia coli*, Rim11 was expressed and purified from *S. cerevisiae* to retain endogenous autophosphorylation on Tyr-199 that is required for its activity (79, 80). We first examined whether Pho85-Pho80 phosphorylates Rim11 and affects its electrophoretic mobility in SDS-PAGE. In the absence of ATP, Rim11-TAP incubated with Pho85-Pho80 migrated at the expected molecular mass of 64 kDa (43-kDa Rim11 + 21-kDa TAP tag) (Fig. 3*A*). In the presence of ATP, however, the kinase-treated Rim11-TAP showed a decrease in its electrophoretic mobility, indicating that Rim11 is phosphorylated by Pho85-Pho80 (Fig. 3*A*).

**Figure 2:**
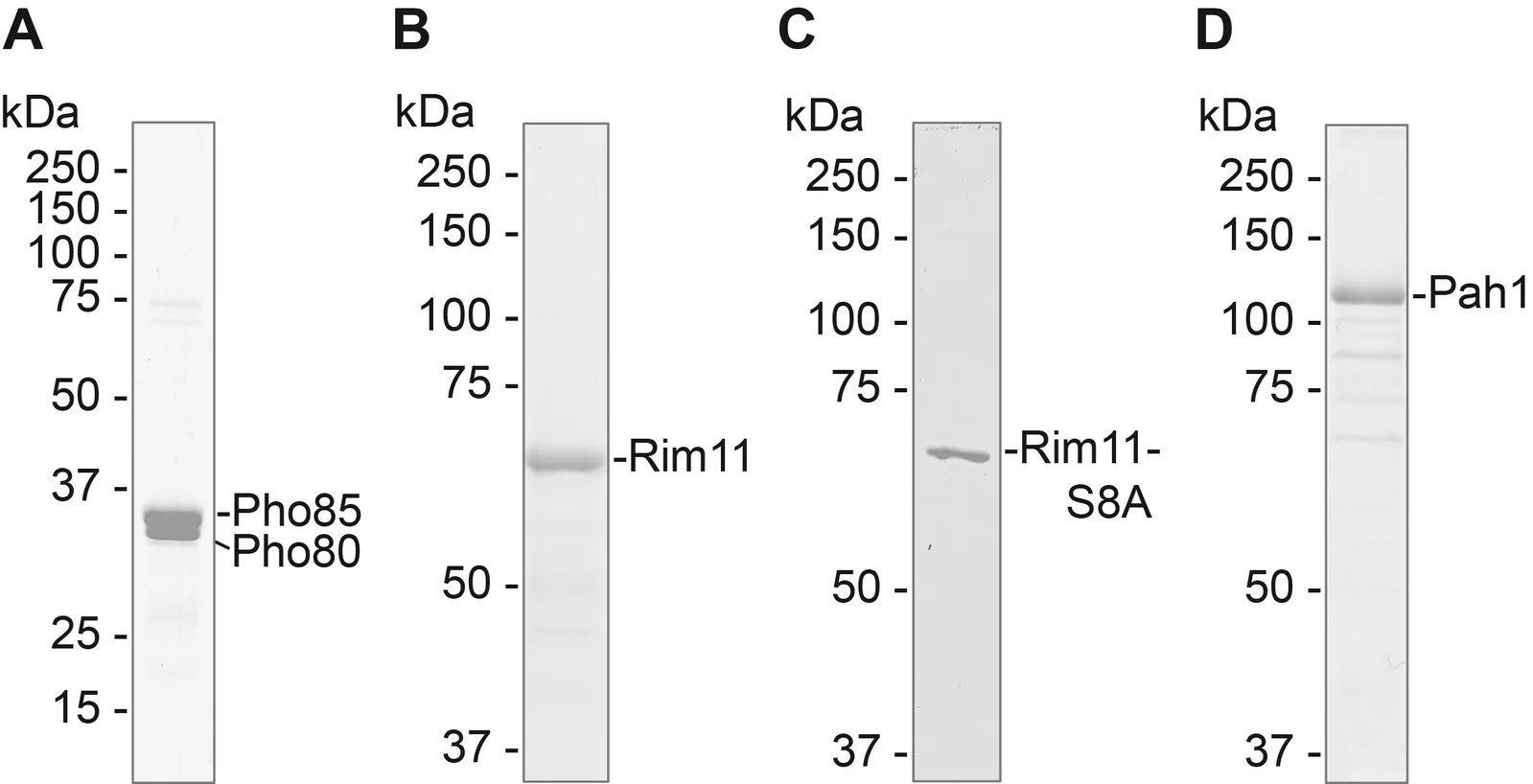
**Purification of the epitope-tagged Pho85-Pho80, Rim11, Rim11-S8A, and Pah1.** Purified preparations of His_6_-tagged Pho85-Pho80 (*A*), TAP-tagged Rim11 (*B*), TAP-tagged Rim11-S8A (*C*), and His_6_-tagged Pah1 (*D*) were subjected to SDS-PAGE (12% and 10% polyacrylamide gels in *A* and *C* and *D*, respectively) and stained with *Coomassie blue*. The positions of the purified proteins and molecular mass standards are indicated. The TAP tag increases the size of Rim11 by 21 kDa, whereas the His_6_ tag increases the sizes of Pho85 and Pah1 by 0.8 kDa and 2.2 kDa, respectively.

**Figure 3:**
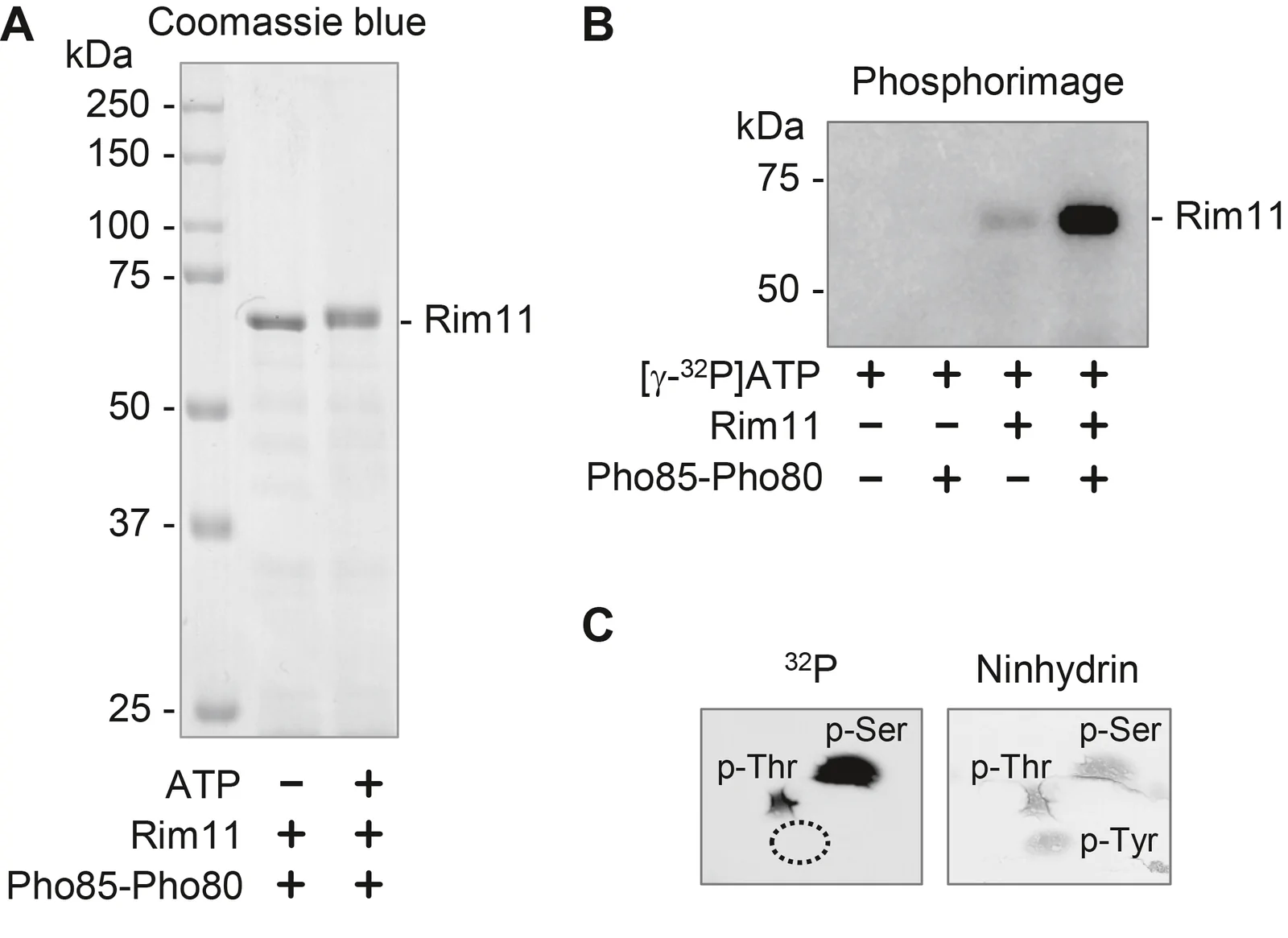
**Pho85-Pho80 phosphorylates Rim11 on serine and threonine residues.***A*, 400 ng Rim11 was incubated for 2 h at 30 °C with 100 ng Pho85-Pho80 in the absence (−) and presence (+) 100 μM ATP. The reaction mixtures were resolved by SDS-PAGE (10% polyacrylamide gel) and subjected to protein staining with *Coomassie blue*. *B*, 100 μM [γ-^32^P]ATP (3000 cpm/pmol) was incubated for 30 min at 30 °C in the absence (−) and presence (+) of 120 ng Rim11 and 10 ng Pho85-Pho80. The reaction mixtures were resolved by SDS-PAGE (10% polyacrylamide gel) and subjected to phosphorimaging. *C*, ^32^P-labeled Rim11 was extracted from an SDS-polyacrylamide gel and hydrolyzed by 6 N HCl for 3 h at 100 °C. The acid hydrolyzate was resolved on a cellulose TLC plate by two-dimensional electrophoresis and subjected to phosphorimaging (*left*). The phosphoamino acid standards phosphoserine (*p-Ser*), phosphothreonine (*p-Thr*), and phosphotyrosine (*p-Tyr*) were visualized by ninhydrin staining (*right*). The absence of ^32^P-labeled p-Tyr is indicated by the *dashed ellipse*. The data in panels *A–C* are representative of three experimental replicates.

To quantify the Rim11 phosphorylation, we examined the incorporation of the radioactive phosphate from [γ-^32^P]ATP into the protein. The ^32^P-labeled Rim11 was resolved from [γ-^32^P]ATP by SDS-PAGE and detected by phosphorimaging (Fig. 3*B*). Compared with the kinase-untreated Rim11, the kinase-treated protein showed an increase in the extent of the phosphate incorporation, confirming that Rim11 is phosphorylated by Pho85-Pho80. The very weak phosphorylation of Rim11 observed in the absence of Pho85-Pho80 is likely to result from the autophosphorylation of the molecules that did not undergo the modification on Tyr-199 *in vivo* (79, 80). The ^32^P-labeled Rim11 produced by Pho85-Pho80 was hydrolyzed and then resolved on a cellulose TLC plate by two-dimensional electrophoresis. The phosphoamino acid analysis showed that the Pho85-Pho80 phosphorylation of Rim11 occurred on the serine and threonine residues with the former accounting for ∼ 90% of the total phosphorylation (Fig. 3*C*). In this analysis, the *in vitro* phosphorylated Rim11 did not show tyrosine phosphorylation, which is attributed to autophosphorylation (79, 80) (Fig. 3*C*).

We further characterized Pho85-Pho80 with Rim11 as a substrate. The kinase activity was dependent on the amount of Pho85-Pho80 and reaction time (Fig. 4, *A* and *B*). In addition, the Pho85-Pho80 activity was dependent on the concentrations of Rim11 and ATP (Fig. 5, *A* and *B*). Moreover, the phosphorylation of Rim11 was nearly abolished when its principal phosphorylation site (Ser-8, see below) was mutated to the unphosphorylable alanine residue (Fig. 4*A*). Altogether, these enzymological properties demonstrate that Rim11 is a *bona fide* substrate of Pho85-Pho80.

**Figure 4:**
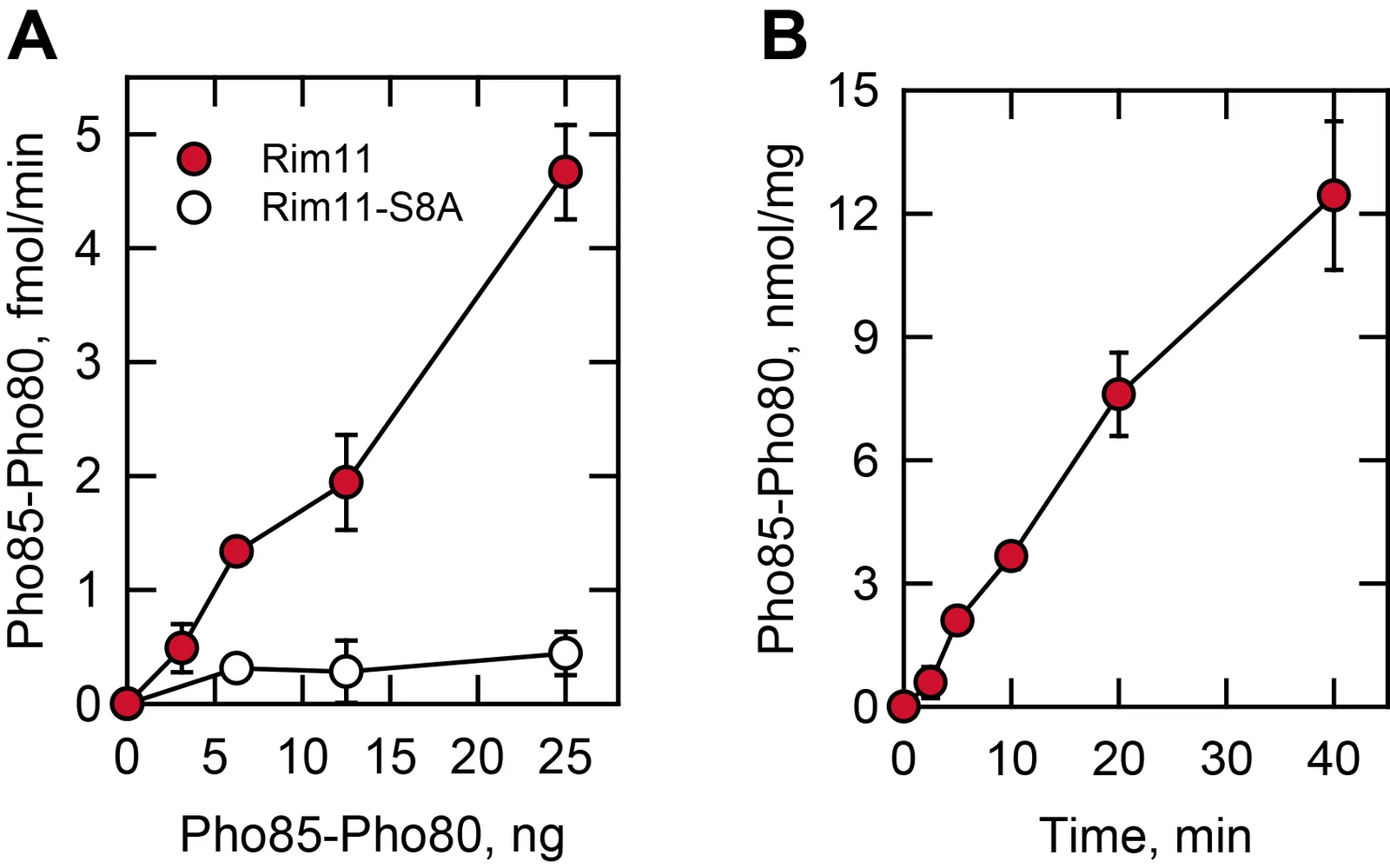
**Pho85-Pho80 kinase activity on Rim11 is dependent on the amount of Pho85-Pho80 and the reaction time.** Rim11 and Rim11-S8A were incubated at 30 °C with Pho85-Pho80 and [γ-^32^P]ATP, subjected to SDS-PAGE (10% polyacrylamide gel), and analyzed by phosphorimaging. The enzyme assay was conducted by varying the amount of Pho85-Pho80 (*A*) and the reaction time (*B*). *A*, 0.2 μM Rim11 or Rim11-S8A/100 μM ATP/10 min; *B*, 0.2 μM Rim11/100 μM ATP/10 ng Pho85-Pho80. The data shown are means ± S.D. (*error bars*) from triplicate assays. Some error bars are hidden behind the circles.

**Figure 5:**
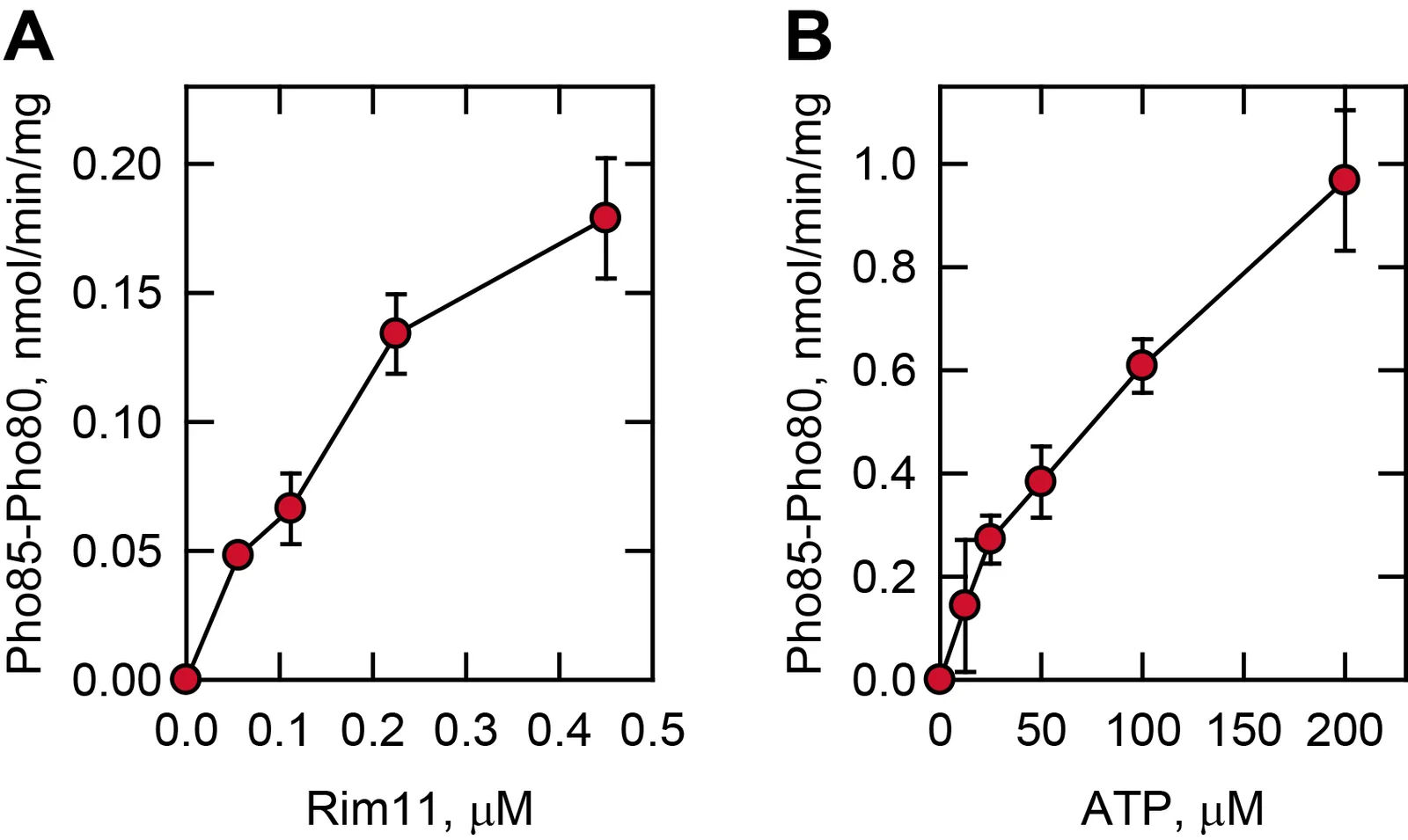
**Pho85-Pho80 kinase activity on Rim11 is dependent on the amounts of Rim11 and ATP.** Rim11 was incubated at 30 °C with Pho85-Pho80 and [γ-^32^P]ATP, subjected to SDS-PAGE (10% polyacrylamide gel), and analyzed by phosphorimaging. The enzyme reaction was conducted by varying the amounts of Rim11 (*A*), and ATP (*B*). *A*, 100 μM ATP/10 ng Pho85-Pho80/10 min; *B*, 0.2 μM Rim11/10 ng Pho85-Pho80/10 min. The data shown are means ± S.D. (*error bars*) from triplicate assays. Some error bars are hidden behind the circles.

### Pho85-Pho80 phosphorylates Rim11 on Ser-8 and Thr-258

To identify the phosphorylated residues of Rim11, we utilized the LC-MS/MS analysis of phosphopeptides derived from the protein by digestion with trypsin, chymotrypsin, and Asp-N (Fig. 6*A*). The phosphopeptides of the unphosphorylated (-*ATP*) Rim11 contained phospho-Tyr-199, indicating the autophosphorylation on the tyrosine residue *in vivo*. For the phosphorylated (+*ATP*) Rim11, principal phosphopeptides containing phospho-Ser-8 were derived from the protein digestion with trypsin, chymotrypsin, and Asp-N, whereas phosphopeptides containing phospho-Thr-258 were derived from the protein digestion with trypsin and Asp-N (Fig. 6*A*). As expected, phosphopeptides containing phospho-Tyr-199 were also present in the phosphorylated sample. These results indicate that the Pho85-Pho80 phosphorylation sites of Rim11 are Ser-8 and Thr-258 with the former being the main site. The positions of these phosphosites within the domain structure of Rim11 are shown in Figure 6*B*.

**Figure 6:**
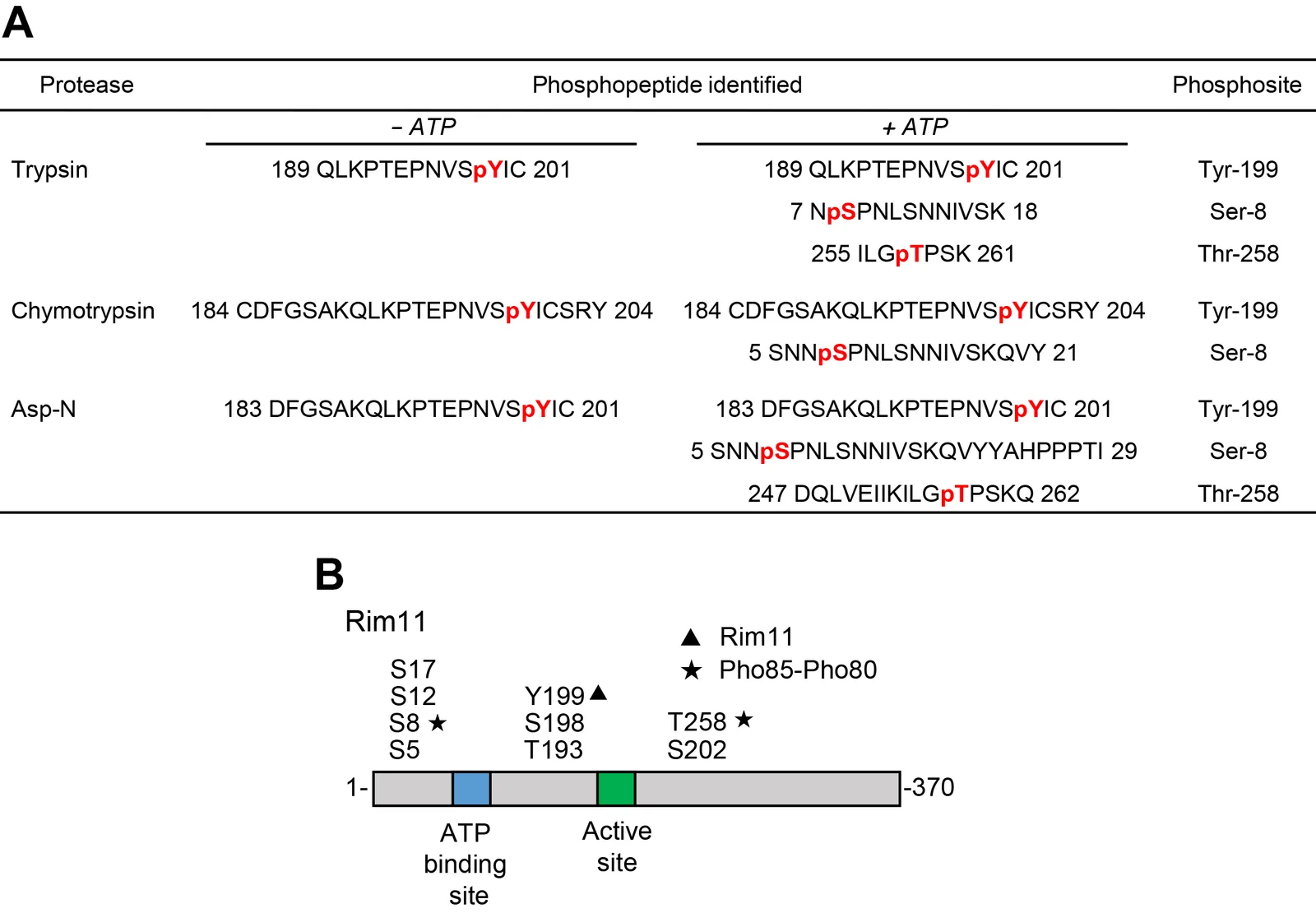
**Pho85-Pho80 phosphorylates Rim11 on Ser-8 and Thr-258.***A*, Rim11 phosphopeptides identified by LC-MS/MS following its phosphorylation by the Pho85-Pho80 complex. The phosphopeptides listed are the most abundant ones identified in the analysis. *B*, the diagram shows the domains, regions, and phosphorylation sites in Rim11. The ATP binding site and Ser/Thr protein kinase active site are required for Rim11 activity. The serine and threonine residues of Rim11 known to be phosphorylated (47, 49, 50, 52, 53, 55, 56, 58, 76, 77, 78) are grouped at their approximate regions. The sites phosphorylated by Pho85-Pho80 (this study) are indicated.

To confirm that Ser-8 and Thr-258 of Rim11 are target sites of phosphorylation by Pho85-Pho80, we utilized the Rim11 peptides RRRNIQSNN**S**PNLSNNI (residues 2-15) and RRRIIKILG**T**PSKQEIC (residues 252-265) that contain the phosphosites Ser-8 and Thr-258, respectively. Three arginine residues were added at the N terminus of each peptide to facilitate its binding to P81 phosphocellulose paper for radioactivity measurement by scintillation counting (82). The phosphorylation of the Rim11 peptides was monitored by following the incorporation of the radioactive phosphate from [γ-^32^P]ATP into the peptide. Pho85-Pho80 catalyzed the phosphorylation of the peptides, and its activity was dependent on the concentration of the peptides (Fig. 7*A*). The Rim11 peptide concentration needed for Pho85-Pho80 activity was in the mM range, which is much higher than the full-length Rim11 concentration in the μM range for the protein kinase activity (Fig. 6*A*). This suggests that the structure of Rim11 is important for phosphorylation. Nonetheless, at the substrate concentration of 0.8 mM, the Pho85-Pho80 activity on the Ser-8 peptide was 9-fold greater when compared with the activity on the Thr-258 peptide (Fig. 7*A*). This result is consistent with the phosphoamino acid analysis, which indicates that 90% of the phosphorylation occurs on the serine residue. The phosphorylation of the Rim11 peptides was abolished by the alanine substitution for Ser-8 (RRRNIQSNN**A**PNLSNNI) or Thr-258 (RRRIIKILG**A**PSKQEIC) (Fig. 6*A*). These results confirmed that Ser-8 and Thr-258 of Rim11 are the phosphorylation sites, and that Ser-8 is the main site of phosphorylation.

**Figure 7:**
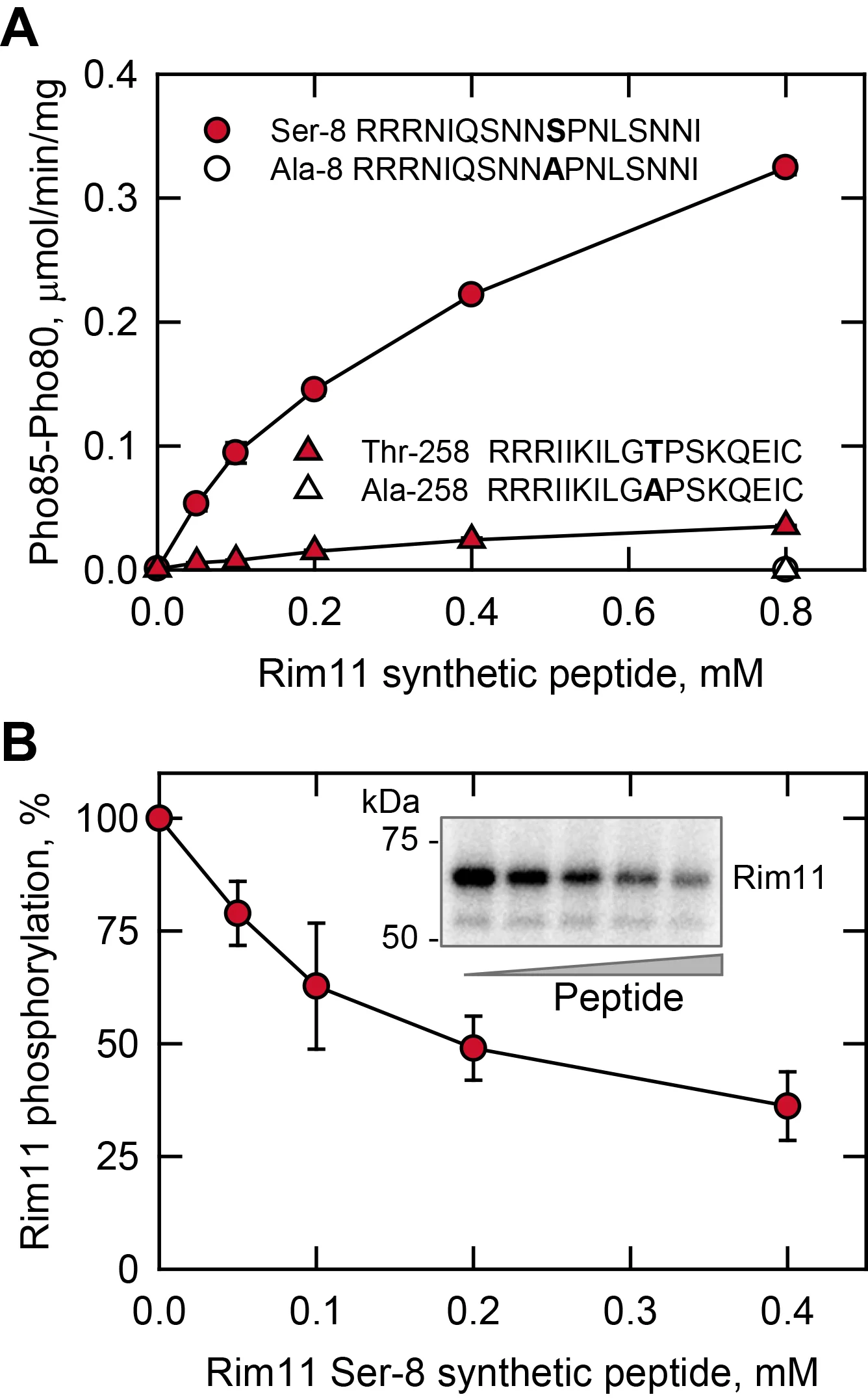
**Pho85-Pho80 phosphorylates the Rim11 synthetic peptides containing Ser-8 and Thr-258; the Rim11 synthetic peptide containing Ser-8 inhibits the phosphorylation of Rim11 by Pho85-Pho80.***A*, 100 ng Pho85-Pho80 was incubated at 30 °C for 10 min with 100 μM [γ-^32^P]ATP (2000 cpm/pmol) and the indicated concentrations of Rim11 peptides. The enzyme reactions were terminated by spotting the mixtures onto the P81 phosphocellulose paper, which was then washed with 75 mM phosphoric acid and subjected to scintillation counting. The bold residue in the peptide sequence designates the Ser-8 and Thr-258 of Rim11 and their Ala substitutions. *B*, 0.14 μM (120 ng) Rim11 was incubated for 30 min with 10 ng Pho85-Pho80 and 100 μM [γ-^32^P]ATP in the absence and presence of the indicated concentrations of the Rim11 Ser-8 peptide. The kinase reaction mixtures were analyzed for the phosphorylation of Rim11 (*insert*). Rim11 was separated from labeled ATP by SDS-PAGE (10% polyacrylamide gel) and subjected to phosphorimaging and ImageQuant analysis. The extent of phosphorylation of Rim11 was set at 100%. The positions of Rim11 and the molecular mass standards are indicated. The data are the averages of three experiments ± S.D. (*error bars*). Some error bars are hidden behind the circles. The phosphorimage shown in *B* (*insert*) is representative of three experiments.

We also examined whether the Ser-8 peptide competes with full-length Rim11 as the substrate of Pho85-Pho80. Rim11 was incubated with the varying amount of the Ser-8 peptide in the presence of Pho85-Pho80 and [γ-^32^P]ATP, and resolved by SDS-PAGE for the phosphorimaging analysis. Quantification of the phosphorylated Rim11 showed that its phosphorylation by Pho85-Pho80 was inhibited by the Ser-8 peptide in a dose-dependent manner (Fig. 7*B*), indicating that the Ser-8 peptide is a competitive inhibitor of Rim11 phosphorylation by Pho85-Pho80. As noted previously, the Rim11 peptides were poor substrates when compared with Rim11, and the concentrations of the Ser-8 peptide needed to inhibit the Rim11 phosphorylation were in the mM range (Fig. 7*B*).

### Pho85-Pho80 stimulates the kinase activity of Rim11 on Pah1

We examined the kinase activity of Rim11 on Pah1 in the linear range (66) with [γ-^32^P]ATP using the Pho85-Pho80-phosphorylated (p-Rim11) form of the enzyme. Following the kinase reaction, Pah1 was resolved by SDS-PAGE, stained with Coomassie blue, and then subjected to phosphorimaging and ImageQuant analysis. Quantification of the radioactive signal showed that the p-Rim11 catalyzed a 6.5-fold greater transfer of radioactive phosphate from [γ-^32^P]ATP into Pah1 when compared with the unphosphorylated form (Fig. 8*A*). To further support that Pho85-Pho80 stimulates Rim11 for its kinase activity on Pah1, we carried out the enzyme assay with unlabeled ATP and evaluated the electrophoretic mobility of Pah1 as its phosphorylation extent (66) (Fig. 8*B*). Within the linear range of the Rim11-mediated phosphorylation of Pah1 (66), p-Rim11 induced a significant reduction in the electrophoretic mobility of Pah1 when compared with the unphosphorylated form of Rim11 (Fig. 8*B*), indicating that Rim11 phosphorylated by Pho85-Pho80 is more active. In contrast to WT Rim11, the S8A mutant did not change the electrophoretic mobility of Pah1 (Fig. 8*B*). These results confirm that the phosphorylation of Rim11 on Ser-8 stimulates the kinase activity on Pah1.

**Figure 8:**
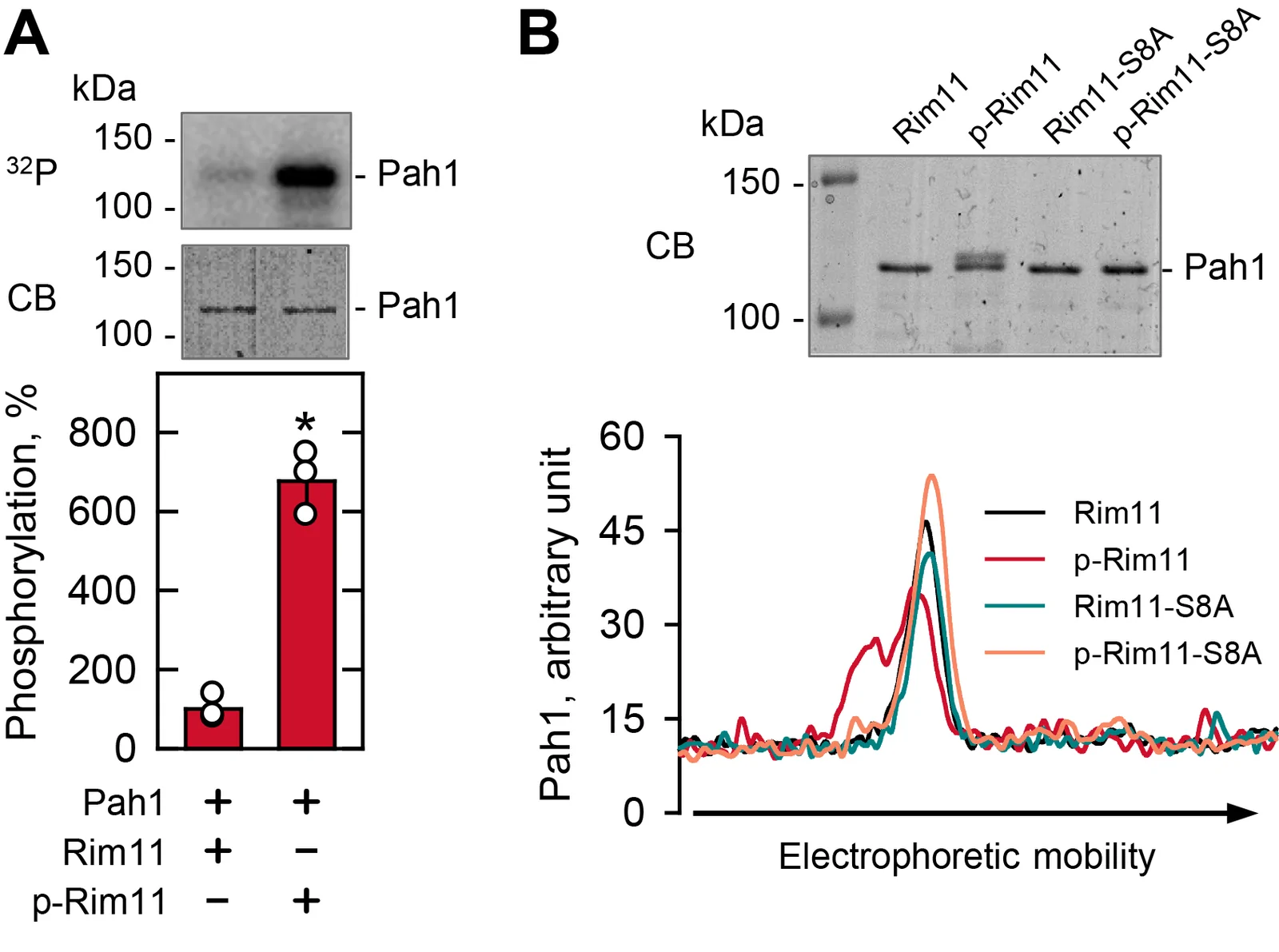
**Phosphorylation of Rim11 by Pho85-Pho80 increases its activity on Pah1.***A*, 3.5 μg of Rim11 or Rim11-S8A was phosphorylated by 100 ng Pho85-Pho80 with 200 μM ATP for 2 h at 30 °C. Following the incubation, the phosphorylated Rim11 (p-Rim11) or Rim11-S8A (p-Rim11-S8A) was separated from Pho85-Pho80 and unreacted ATP by filtration through a Pierce Concentrator. *A*, 0.4 μg Pah1 was incubated with 100 μM [γ-^32^P]ATP (6000 cpm/pmol) and 50 ng Rim11 or p-Rim11 for 30 min at 30 °C. The ^32^P-labeled Pah1 was resolved by SDS-PAGE (10% polyacrylamide gel), stained with *Coomassie blue* (*CB*), and then subjected to phosphorimaging (^*32*^*P*) and ImageQuant analysis. The phosphorimage and *Coomassie blue* images (*upper*) are representative of three experiments, whereas the bar chart (*lower*) shows the individual data points of the three experiments ± S.D. (*error bars*). The phosphorylation of Pah1 by unphosphorylated Rim11 was set at 100%. ∗, *p* < 0.05 phosphorylation of Pah1 by p-Rim11 *versus* phosphorylation by Rim11. *B*, 1 μg Pah1 was incubated with 100 μM ATP and 50 ng Rim11 or p-Rim11 (*left*) or 50 ng Rim11-S8A or p-Rim11-S8A (*right*) for 30 min at 30 °C Pah1 was resolved by SDS-PAGE (6% polyacrylamide gel) and stained with *Coomassie blue* (*upper*). The densitograms of Pah1 along its electrophoretic migration was produced by the line graph function of ImageQuant software (*lower*). The data are representative of three experimental replicates. The positions of Pah1 and molecular mass standards in *A* and *B* are indicated.

### Rim11 and Pho85-Pho80 regulates Pah1 for the expression of Cho1 PS synthase

The committed step in *de novo* membrane phospholipid synthesis in yeast is catalyzed by the *CHO1*-encoded PS synthase (3, 6, 8, 83, 84) (Fig. 1*A*). PS synthase catalyzes the displacement of CMP from CDP-DAG to form PS (83, 84), which is subsequently converted into phosphatidylethanolamine and phosphatidylcholine (Fig. 1). Expression of *CHO1*, one of the UAS_INO_-containing genes that is regulated by *via* the Henry (Ino2–Ino4/Opi1) regulatory circuit (3, 6, 8, 26, 85) (Fig. 1*A*), is normally repressed during the stationary phase (27) (Fig. 9*A*, WT *PAH1* expressed in *pah1*Δ cells). As previously reported (27, 86), the growth-dependent regulation of *CHO1* expression is disrupted in *pah1*Δ and *pah1*Δ *nem1*Δ mutants (Fig. 9*A* and *B*, vector controls), underscoring the role of Pah1 in controlling the Opi1-mediated repression of *CHO1* (27) (Fig. 1*A*). For example, in the stationary phase, the Cho1 amount was ∼3-fold higher in the *pah1*Δ mutant than in the mutant expressing WT *PAH1* (Fig. 9*C*). Introduction of WT *PAH1* into the *pah1*Δ *nem1*Δ mutant did not restore normal regulation of *CHO1* (Fig. 9*D*), reflecting the requirement of the Nem1-Spo7 complex for proper Pah1 function (70, 73, 86). As expected (45, 66), Pah1 was detected by Western blot analysis in extracts from exponential-phase cells but decreased in abundance during stationary phase (Fig. 9, *A* and *B*), consistent with its proteasomal degradation (67). In contrast, Pah1 abundance in the exponential-phase *pah1*Δ *nem1*Δ is stabilized due to its hyperphosphorylated state in the absence of the Nem1-Spo7 complex (45, 66, 68) (Fig. 9*B*).

**Figure 9:**
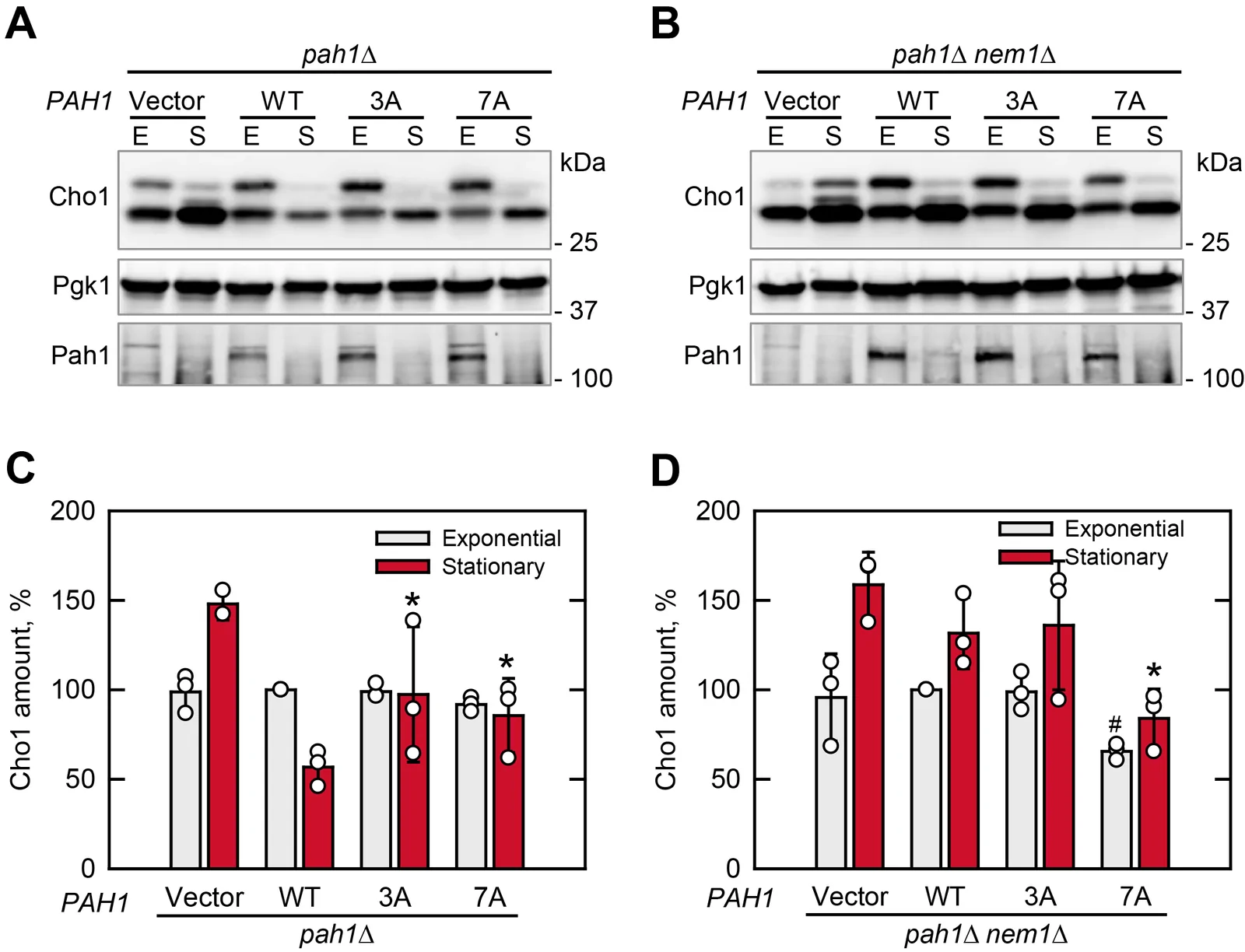
**Phosphorylation-deficient site mutations in Pah1 affect the regulation of *CHO1* expression.** The *pah1*Δ (SS1026) (*A*) and *pah1*Δ *nem1*Δ (SS1132) (*B*) cells expressing the WT, Rim11 phosphorylation-deficient T163A/T164A/S602A (*3A*), or Pho85-Pho80 phosphorylation-deficient S110A/S114A/S168A/S602A/T723A/S744A/S748A (*7A*) forms of Pah1 were grown at 30 °C in SC-Leu medium to the exponential (*E*) and stationary (*S*) phases. Cell extracts were prepared, resolved by SDS-PAGE, and transferred to a polyvinylidene difluoride membrane. The membrane was probed for Cho1, Pgk1 (loading control), and Pah1 with rabbit anti-Cho1, mouse anti-Pgk1, and rabbit anti-Pah1, antibodies, respectively. Sample loading was normalized to total protein. The positions of Cho1, Pgk1, and Pah1 are indicated with molecular mass standards. The *upper* and *lower* signals of Cho1 are its phosphorylated and unphosphorylated forms, respectively (112). The Western blots are representative of three independent experiments. *C* and *D*, the relative amounts of Cho1 (phosphorylated and unphosphorylated forms combined) were determined by ImageQuant analysis of the triplicate experiments ± SD (*error bars*). The individual data points are also shown. ^#^, *p* < 0.05 *versus* WT in exponential phase cells. ∗, *p* < 0.05 *versus* WT in stationary phase cells.

We tested the hypothesis that the phosphorylation state of Pah1, as mediated by Rim11 and Pho85-Pho80, regulates *CHO1* expression. To address this, we examined the *CHO1* expression of the *pah1*Δ and *pah1*Δ *nem1*Δ mutants transformed with *PAH1* alleles lacking phosphorylation sites for Rim11 (T163A/T164A/S602A; 3A) and Pho85-Pho80 (S110A/S114A/S168A/S602A/T723A/S744A/S748A; 7A). In the stationary phase, the phosphorylation-deficient Pah1 mutants (Rim11 and Pho85-Pho80 sites) disrupted normal regulation of *CHO1* expression. Specifically, Cho1 levels in *pah1*Δ cells expressing the Pah1-3A or -7A were 1.7-fold higher than in cells expressing WT *PAH1* (Fig. 9*C*).

To determine whether the regulation depends on the dephosphorylation of Pah1, we expressed the 3A and 7A alleles in the *pah1*Δ *nem1*Δ mutant, which lacks the Nem1-Spo7 complex. In this genetic background, *CHO1* expression in the transformant expressing the 3A allele resembled that of transformants expressing WT *PAH1* (Fig. 9*D*), reflecting the loss of *CHO1* regulation when the Nem1-Spo7 complex is lacking whether or not Pah1 is phosphorylated by Rim11. However, in the transformant expressing the 7A allele, the *CHO1* expression was reduced by 1.6-fold relative to the WT control (Fig. 9*D*). These findings indicate that the 7A mutant can bypass the requirement of dephosphorylation to regulate *CHO1* expression.

The yeast transformants expressing the phosphorylation-deficient alleles of *PAH1* were also analyzed for lipid content by steady-state labeling with [2-^14^C]acetate. As described previously (7, 87), the amount of TAG in cells lacking Pah1 was greatly reduced in both the exponential and stationary phases of growth (Fig. 10, *A* and *B*, vector controls). Expression of WT *PAH1* in *pah1*Δ cells complemented the defect in lipid synthesis. In these cells, the amounts of TAG and phospholipid were ∼ 3-fold higher and ∼ 2-fold lower, respectively, in stationary phase cells when compared with that of exponential phase cells (Fig. 10*A*, WT *PAH1* expressed in *pah1*Δ cells). This observation is consistent with the stationary phase-mediated induction of Pah1 function (35) and repression of *CHO1* expression (27) (Fig. 9). Aside from a modest reduction in TAG content (1.25-fold) caused by the 7A allele, the lipid content of *pah1*Δ cells expressing the 3A or 7A *PAH1* allele was comparable to that of the WT control (Fig. 10*A*).

**Figure 10:**
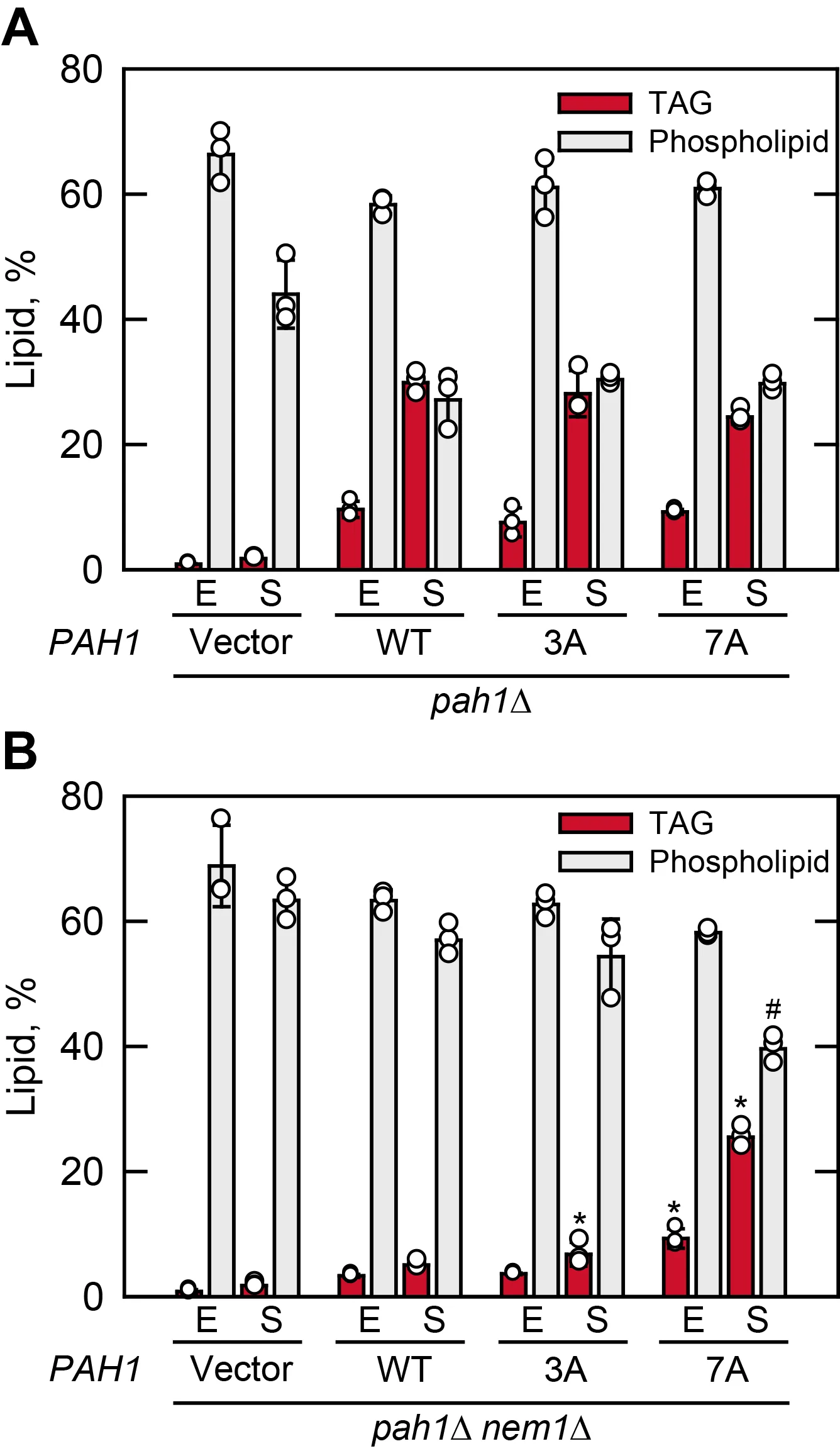
**Phosphorylation-deficient site mutations in Pah1 affect lipid content.** The *pah1*Δ (SS1026) (*A*) and *pah1*Δ *nem1*Δ (SS1132) (*B*) cells expressing the WT, Rim11 phosphorylation-deficient T163A/T164A/S602A (*3A*), or Pho85-Pho80 phosphorylation-deficient S110A/S114A/S168A/S602A/T723A/S744A/S748A (*7A*) forms of Pah1 were grown at 30 °C to the exponential and stationary phases in 1 ml of SC-Leu medium containing [2-^14^C]acetate (1 μCi/ml). Lipids were extracted, separated by one-dimensional TLC, and subjected to phosphorimaging, followed by ImageQuant analysis. The percentages shown for TAG and phospholipids were normalized to the total ^14^C-labeled chloroform-soluble fraction. The data are means ± SD (*error bars*) from three separate experiments. The individual data points are also shown. ^#^, *p* < 0.05 *versus* WT in exponential phase cells. ∗, *p* < 0.05 *versus* WT in stationary phase cells. TAG, triacylglycerol.

Lack of the Nem1-Spo7 complex in *pah1*Δ *nem1*Δ cells obviated the complementation of the lipid content defect by WT *PAH1* (Fig. 10*B*). In addition, the growth-phase regulation of lipid content was largely lost in these cells, further underscoring the critical role of Pah1 dephosphorylation by the Nem1-Spo7 complex. The expression of the phosphorylation-deficient 3A and 7A alleles in *pah1*Δ *nem1*Δ cells resulted in 1.4-fold and 5-fold increases, respectively, in TAG content in the stationary phase (Fig. 10*B*). These results are consistent with those observed for the growth phase regulation of *CHO1* expression by the phosphorylation state of Pah1 (Fig. 9). The Pho85-Pho80-mediated phosphorylation ensures the dependence of Pah1 function on Nem1-Spo7 (32, 45, 70).

## Discussion

PA phosphatase Pah1 (7), which regulates the PA/DAG balance for synthesis of lipids and a host of physiological processes including the expression of phospholipid synthesis genes (1, 2, 3, 4, 5, 6), is highly phosphorylated on Ser/Thr residues by multiple protein kinases (32, 47, 48, 49, 50, 51, 52, 53, 54, 55, 56, 57, 58) (Fig. 1*B*). The phosphorylation of Pah1, along with its dephosphorylation by the Nem1-Spo7 protein phosphatase, plays a role in the control of the enzyme location, activity, and stability (46). Many of the Pah1 phosphosites are targets of two or more protein kinases (46), most notably Ser-602, which is phosphorylated by Pho85-Pho80 (45) and Rim11 (66) as well as by Cdc28-cyclin B (44) and casein kinase I (63) (Fig. 1*B*). Employing multiple protein kinases that act on the same phosphosite provides a level of redundancy in cellular signaling, ensuring the maintenance of the phosphorylation-mediated regulation (88, 89, 90). Protein kinases that are active at different times allow for the fine-tuned control of a specific site phosphorylation depending cellular conditions (44, 45, 62). The phosphorylation state of a particular site can be a point of convergence for different signaling pathways to provide the intricate control of enzyme function (89, 90). Our current work expands the understanding of the mechanism of Pah1 phosphorylation catalyzed by Pho85-Pho80 and Rim11.

Pho85, the *S. cerevisiae* homolog of mammalian Cdk5, was first known for its role in the regulation of phosphate metabolism (91, 91, 92). It is now recognized as a multifunctional cyclin-dependent protein kinase involved in a variety of signal transduction pathways affecting cell cycle progression, nutrient metabolism, and lipid synthesis (45, 92, 93, 94, 95, 96, 97). Rim11, the *S. cerevisiae* homolog of mammalian glycogen synthase kinase-3β, was originally identified as a protein kinase required for signal transduction during entry into meiosis (98) as it promotes the formation of the Ime1-Ume6 transcriptional activator complex by phosphorylating each subunit (99, 100, 101). The kinase was later shown to be involved in the regulation of lipid synthesis (66) and glutathione-induced stress (102). Pho85-Pho80 (45) and Rim11 (66) phosphorylate Pah1 on multiple sites in a hierarchical manner to sequester the enzyme in the cytosol and reduce its catalytic activity (45, 66). The expression of the phosphorylation-deficient forms of Pah1 in *pah1*Δ *nem1*Δ cells, which lack the Nem1-Spo7 complex, results in an increase in TAG content (44, 45, 66) (Fig. 10*B*).

This work showed that Pho85-Pho80 phosphorylates Rim11 to stimulate its kinase activity on Pah1. Rim11 is mainly phosphorylated on Ser-8 (90% of the phosphorylation), which is within the minimal (Ser/Thr)-Pro motif targeted for phosphorylation by proline-directed protein kinases (81). The serine phosphorylation is responsible for the stimulation of the Rim11 kinase activity on Pah1. The Pho85-Pho80-mediated stimulation of Rim11 activity demonstrates the cascade of protein kinase reactions to phosphorylate Pah1 (Fig. 11). Pho85-Pho80 and Rim11 phosphorylate Pah1 on Ser-602 as a common target site in addition to other distinct residues targeted by these protein kinases (45, 66) (Fig. 1*B*). The S602A mutation alone imparts a partial defect in the Nem1-Spo7-dependent regulation of Pah1 function in lipid synthesis and cell physiology, whereas the phosphosite mutation combined with the mutations of other sites phosphorylated by Pho85-Pho80 and Rim11 elicits a stronger effect on the regulation of the protein function (44, 45, 66). The physiological conditions that affect the direct and/or indirect phosphorylation of Pah1 by Pho85-Pho80 and Rim11 are unclear, but we speculate that they are governed by growth phase, nutrient availability, and stress conditions that trigger alterations in lipid synthesis (3, 6, 8, 102, 103).

**Figure 11:**
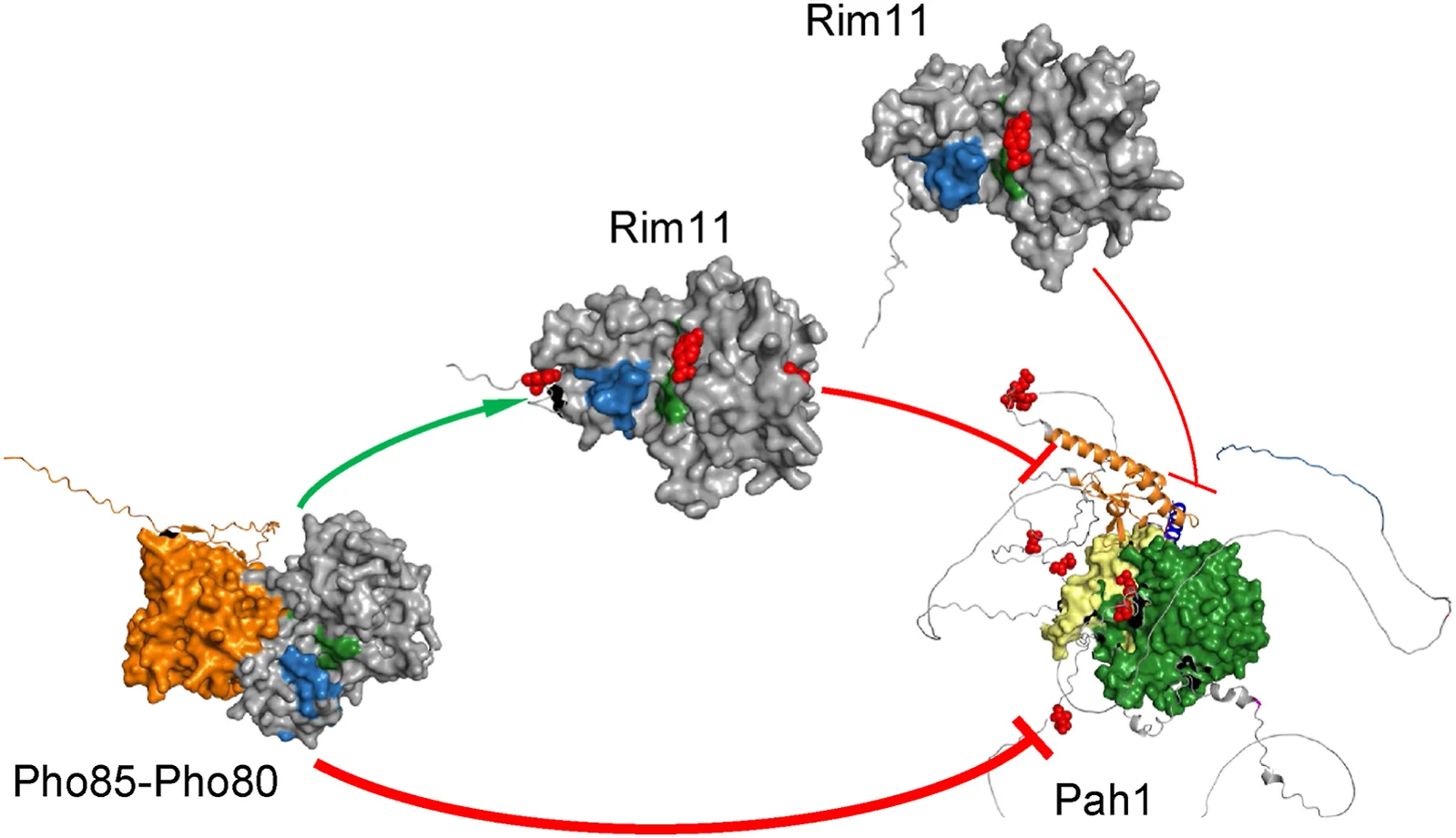
**Model for the phosphorylations of Pah1 by Pho85-Pho80 and Rim11.** The figure shows the AlphaFold3 structures of Pho85-Pho80, Rim11, and Pah1. The active sites and ATP binding sites, respectively, in Pho85 and Rim11 are shown in *green* and *blue*, respectively. The *red circles* depict the phosphorylated residues in Rim11 as mediated by autophosphorylation (Tyr-199) and Pho85-Pho80 (Ser-8 and Thr-258), and phosphorylated residues in Pah1 as mediated by Rim11 (Thr-163, Thr-164, and Ser-602) and Pho85-Pho80 (Ser-110, Ser-114, Ser-168, Ser-602, Thr-723, Ser-744, and Ser-748). Pho85-Pho80 phosphorylates and stimulates the protein kinase activity of Rim11 (*green arrow*). The Pho85-Pho80-and Rim11-mediated phosphorylation negatively regulates Pah1 function. The thickness of the *blunted red lines* denotes the relative contribution of each protein kinase to this regulatory effect. Crosstalk between protein kinases regulates lipid synthesis.

Pah1, being a PA phosphatase (7, 104), plays a major role in regulating the expression of phospholipid synthesis genes (*e*.*g*., *INO1*, *INO2*, *CHO1*, and *OPI3*) that contain the UAS_INO_ element in the promoter (27, 29, 32) (Fig. 1*A*). In this regulation, the protein–lipid interaction of Opi1 and PA controls the nuclear localization of the transcriptional repressor (105, 106, 107, 108) (Fig. 1*A*). The higher level of PA, as mediated by lower PA phosphatase activity, increases the amount of Opi1 interacting with PA and Scs2 at the ER membrane, which prevents the transcriptional repressor from entering the nucleus and allows for the function of Ino2-Ino4 to drive transcription (3, 6, 8, 26, 109, 110) (Fig. 1*A*, *left*). In contrast, the lower level of PA, as mediated by higher PA phosphatase activity, increases the production of free Opi1, which enters the nucleus for its inhibitory interaction with Ino2 and thereby represses the expression of phospholipid synthesis genes (3, 6, 8, 26, 109, 110, 111) (Fig. 1*A*, *right*). In line with the regulatory mechanism, the *pah1*Δ mutant, which lacks Pah1 PA phosphatase activity, exhibits a ∼4-fold increase in the cellular level of PA and an increase in the *CHO1* expression as scored by the levels of Cho1 (27) (Fig. 9).

We investigated whether *CHO1* expression is regulated through the Rim11-and Pho85-Pho80-dependent phosphorylations of Pah1. To address this, we analyzed cells expressing phosphorylation-deficient Pah1 mutants lacking Rim11 sites (T163A/T164A/S602A; 3A) or Pho85-Pho80 sites (S110A/S114A/S168A/S602A/T723A/S744A/S748A; 7A). Our results indicated that phosphorylation of Pah1 by both Rim11 and Pho85-Pho80 are required for the repression of *CHO1* in the stationary phase of growth. However, their roles are not equivalent, particularly when dephosphorylation by the Nem1–Spo7 complex is considered. This distinction was reflected in the ability of Pah1 with the 7A mutations, but not with the 3A mutations, to bypass the requirement for Nem1-Spo7 complex-mediated dephosphorylation. Moreover, the regulatory effect of Pah1 phosphorylation by Pho85-Pho80 was stronger than that mediated by Rim11. These findings, however, do not fully account for the potential hierarchical phosphorylation of Pah1 on the remaining, nonmutated sites (46). It remains unclear whether the specific Rim11-and Pho85-Pho80-targeted phosphosites mutated here influence the phosphorylation status of Pah1 on other sites, thereby affecting its regulatory function on *CHO1* expression in the presence or absence of Nem1-Spo7.

Our data suggest that the regulatory effects of Rim11 on Pah1 are enhanced by phosphorylation mediated by the Pho85-Pho80 kinase complex. To investigate this possibility, we attempted to analyze *CHO1* expression in *rim11*Δ cells expressing the phosphorylation-deficient *RIM11*-S8A allele. However, as noted previously (66), the slow-growth phenotype associated with the *rim11*Δ background precluded meaningful experimentation. Nonetheless, analysis of cells expressing Pah1 variants containing phosphorylation-deficient mutations at Rim11 target sites indicated that the predominant regulation of *CHO1* expression and lipid synthesis occurs through Pho85-Pho80-mediated phosphorylation rather than through Rim11 itself (Fig. 11).

Beyond its role in Opi1 localization through Pah1 phosphorylation, Pho85-Pho80 appears to control the function of the transcriptional repressor in the nucleus (97). Through direct interaction, Pho85-Pho80 prevents the transcriptional repressor from interacting with Ino2 allowing for transcriptional activation by the Ino2-Ino4 complex (97). Thus, Pho85-Pho80 exerts a negative regulatory effect on Opi1 by controlling its nuclear localization indirectly and by controlling its inhibitory protein–protein interaction directly. The dual-mode regulation of Opi1 by Pho85-Pho80 ensures the transcription of the UAS_INO_-containing phospholipid synthesis genes in response to environmental cues activating the protein kinase.

In summary, this work showed that Rim11 is directly phosphorylated by the Pho85-Pho80 complex at Ser-8, a modification that enhances the ability of the kinase to phosphorylate Pah1. Mutational analyses further demonstrated that the phosphorylation state of Pah1, governed by Pho85-Pho80 and Rim11, regulates the expression of the phospholipid-synthesizing enzyme Cho1 PS synthase. Together, these results suggest a coordinated kinase cascade that controls Pah1 function in lipid synthesis.

## Experimental procedures

### Reagents

DNA size ladders, molecular mass protein standards, Bradford protein assay, and reagents for electrophoresis and Western blotting were from Bio-Rad. Clontech supplied carrier DNA for yeast transformations and Difco Laboratories was the source of growth media. Rim11 peptides were synthesized by EZBiolab. InstantBlue (Coomassie blue) protein stain was from Expedeon. IgG-Sepharose, Q-Sepharose, polyvinylidene difluoride membrane, and the chemifluorescence Western blotting detection kit were from GE Healthcare. MilliporeSigma was the source of ATP, bovine serum albumin, cellulose TLC plates, phosphoamino acid standards, Ponceau S stain, Triton X-100, and protease and phosphatase inhibitors. Radiochemicals and scintillation counting supplies were purchased from Revvity and National Diagnostics, respectively. Pierce was the source of mass spectrometry grade proteases and strong anion exchange spin columns. Q5 site-directed mutagenesis kit and other reagents for DNA manipulations were from New England Biolabs. Qiagen supplied nickel-nitrilotriacetic acid agarose resin and the DNA gel extraction and plasmid purification kits. P81 phosphocellulose paper was purchased from Whatman. All other chemicals were reagent grade.

### Antibodies

Rabbit polyclonal antibodies were generated against the amino acid sequence TSIDKEFKKLSVSKAGA (residues 778-794) of Pah1 (44) and MVESDEDFAPQEFPH (residues 1-15) of Cho1 (112) from *S. cerevisiae*. The IgG fraction of the antiserum (113) was used for immunoblot analyses. MilliporeSigma supplied goat anti-mouse IgG antibody conjugated with alkaline phosphatase (product number A3562, lot number SLBG1482V). Abcam was the source of mouse anti-Pgk1 antibody (product number ab113687, lot number 2101050637). Thermo Fisher Scientific provided goat anti-rabbit IgG antibody conjugated with alkaline phosphatase (product number 31340, lot number NJ178812).

### Strains and plasmids

The strains used in this study are listed in Table 1. The *S. cerevisiae* strain W303-1A was used for the overexpression and purification of the WT and phosphorylation-deficient S8A forms of Rim11. The *S. cerevisiae* mutants *pah1*Δ*::TRP1* (strain SS1026) and *pah1*Δ*::TRP1 nem1*Δ*::HIS3* (strain SS1132) are derivatives of RS453 (29, 44) and were used for low-copy expression of the WT and phosphorylation-deficient (Rim11 or Pho85-Pho80 sites) forms of Pah1. *E. coli* strain DH5α (114) was used for the propagation of plasmids, whereas strains BL21(DE3) and BL21(DE3)pLysS were used for the expressions and purifications of His_6_-tagged Pho85-Pho80 and His_6_-tagged Pah1, respectively.

**Table 1:** Strains and plasmids used in this study

Strain or plasmid	Genotype or relevant characteristics	Source or reference
Strain		
*S*. *cerevisiae*		
W303-1A	*MAT*a *ade2*-*1 can1*-*100 his3*-*11*,*15 leu2*-*3112 trp1*-*1 ura3*-*1*	(132)
RS453	MATα ade2-1 his3-11,15 leu2-3112 trp1-1 ura3-52	(133)
SS1026	*pah1*Δ*::TRP1* derivative of RS453	(29)
SS1132	*pah1*Δ*::TRP1 nem1*Δ*::HIS3* derivative of RS453	(44)
BY4741-*RIM11*-TAP	TAP-tagged *RIM11* strain	Thermo Fisher Scientific
*E. coli*		
DH5α	F^-^ ϕ80d*lacZ*ΔΜ15Δ (*lacZYA*-*argF*)U169 *deoR rec*A1 *end*A1 *hsd*R17 (*r*_k_^-^*m*_*k*_^+^) *pho*A *sup*E44 λ^−^*thi*-1 *gyr*A96 *rel*A1	(114)
BL21 (DE3)pLysS	F^-^*ompT hsdS*_*B*_ (*r*_*B*_^-^*m*_*B*_^-^) *gal dcm* (DE3) pLysS	Novagen
BL21 (DE3)	F^-^*ompT hsdS*_*B*_ (*r*_*B*_^-^*m*_*B*_^-^) *gal dcm* (DE3)	Invitrogen
Plasmid		
pYES2	High-copy *E. coli*/yeast shuttle vector with *URA3* and the *GAL1* promoter	Thermo Fisher Scientific
pSK001	*RIM11*-TAP coding sequence inserted into pYES2	(66)
pTC001	*RIM11* (S8A) derivative of pSK001	This study
pRS415	Low-copy *E. coli*/yeast shuttle vector with *LEU2*	(134)
pGH315	*PAH1* inserted into pRS415	(44)
pSK007	*PAH1* (T163A/T164A/S602A) derivative of pGH315	(66)
pHC204	*PAH1* (S110A/S114A/S168A/S602A/T723A/S744A/S748A) derivative of pGH315	(44)
EB1164	*PHO85*-His_6_ inserted into pQE-60	(81)
EB1076	*PHO80* inserted into pSBETA	(81)
pET-15b	*E. coli* expression vector with N-terminal His_6_-tag fusion	Novagen
pGH313	*PAH1* coding sequence inserted into pET-15b	(7)

The plasmids used in this study are listed in Table 1. pTC001, which contains the coding sequence of *RIM11*-S8A-TAP inserted into pYES2 (66), was generated from pSK001 using the Q5 site-directed mutagenesis kit. pSK001 and pTC001 were used for the overexpression and purification of the WT and phosphorylation-deficient S8A forms of TAP-tagged Rim11. pSK007 (66) and pHC204 (44) were used for the low-copy expression of Pah1 (T163A/T164A/S602A) and Pah1 (S110A/S114A/S168A/S602A/T723A/S744A/S748A) deficient in phosphorylation by Rim11 and Pho85-Pho80, respectively. EB1164/EB1076 and pGH313 were used for the overexpression and purification of the Pho85-Pho80 complex (81) and Pah1 (7), respectively. Standard methods were used for the isolation of chromosomal and plasmid DNA, for the digestion and ligation of DNA, and for the PCR amplification of DNA (114, 115, 116). Plasmid constructs were confirmed by restriction enzyme digestion and DNA sequencing. Plasmid transformations of yeast (117) and *E*. *coli* (114) were performed by standard methods.

### Growth conditions

*S. cerevisiae* and *E. coli* cells were cultured using standard methods (114, 115). Solid media contained 2 and 1.5% agar for the growth of *S. cerevisiae* and *E. coli*, respectively. Growth in liquid medium was monitored by absorbance at 600 nm using the Thermo Scientific BioMate 3S spectrophotometer. *S. cerevisiae* transformants carrying plasmids were grown in synthetic dropout medium, which lacks a specific amino acid or nucleic acid base for plasmid selection. For galactose-induced overexpression of Rim11 and Rim11 (S8A), W303-1A transformants harboring plasmid pSK001 or pTC001 were inoculated into 250 ml of SC-Ura/2% glucose medium to the final cell density of A_600_ ∼ 0.1 and grown to saturation at 30 °C. The saturated culture was harvested by centrifugation at 1500*g* for 10 min, and the cell pellet was resuspended (A_600_ ∼ 0.4) in 2 L of SC-Ura/1% raffinose/2% galactose medium and incubated for 14 h (A_600_ ∼ 1.0) with shaking at 250 rpm. *E*. *coli* transformants bearing plasmids were grown in lysogeny brothLB medium (1% tryptone, 0.5% yeast extract, 1% NaCl, pH 7.0) containing antibiotics for the plasmid selection. For the IPTG-induced overexpression of His_6_-tagged Pho85-Pho80 and Pah1, *E. coli* BL21(DE3) transformants harboring EB1164/EB1076 and those containing pLysS/pGH313 were grown at 37 °C to A_600_ ∼ 0.5 in 500 ml of lysogeny broth medium containing ampicillin (100 μg/ml)/kanamycin (50 μg/ml) and ampicillin (100 μg/ml)/chloramphenicol (34 μg/ml), respectively. The cultures were cooled down to RT and incubated for 1 h with 1 mM isopropyl-*β*-D-thiogalactoside (7). For the analyses of lipids and Cho1 amount, the *S. cerevisiae* transformants were grown in SC-Leu medium.

### Preparation of *S. cerevisiae* cell extracts

All steps were performed at 4 °C. *S. cerevisiae* cultures were harvested by centrifugation at 1500*g* for 5 min. The collected cells were washed with water and resuspended in 50 mM Tris-HCl (pH 7.5) buffer containing 0.3 M sucrose, 10 mM 2-mercaptoethanol, 1 mM EDTA, 0.5 mM phenylmethylsulfonyl fluoride, 1 mM benzamidine, 5 μg/ml aprotinin, 5 μg/ml leupeptin, and 5 μg/ml pepstatin. The cell suspensions were added with glass beads (0.5-mm diameter), and the mixture was then subjected to five repeats of 1-min burst and 2-min cooling using a BioSpec Products Mini-Beadbeater-16 (118). The disrupted cells were centrifuged at 1500*g* for 10 min to separate cell extracts (supernatant) from unbroken cells and cell debris (pellet).

### Purification of enzymes

His_6_-tagged Pho85-Pho80 complex was purified from *E. coli* cells expressing the plasmids EB1164 and EB1076 by affinity chromatography with nickel-nitrilotriacetic acid-agarose (81). TAP-tagged Rim11 and Rim11-S8A were purified from *S. cerevisiae* cells expressing plasmids pSK001 and pTC001, respectively, by affinity chromatography with IgG-Sepharose (66) and eluted with 50 mM glycine (pH 3.0), followed by neutralization with 1M Tris-HCl (pH 8.0) (63, 65). His_6_-tagged Pah1 was purified from *E. coli* cells expressing the plasmid pGH313 by affinity chromatography with nickel-nitrilotriacetic acid-agarose (7), followed by ion exchange chromatography with Q-Sepharose (70). The purified enzyme preparations were stored at – 80 °C.

### Protein kinase assays

Pho85-Pho80 (45) activity was measured at 30 °C by following the incorporation of radioactive phosphate from [γ-^32^P]ATP into Rim11 in a total volume of 20 μl. The reaction mixture contained 50 mM Tris-HCl (pH 7.5), 10 mM MgCl_2_, 2 mM dithiothreitol, 100 μM [γ-^32^P]ATP (∼3000 cpm/pmol), and the indicated concentrations of Rim11. Rim11 (66) activity was measured at 30 °C by following the incorporation of radioactive phosphate from [γ-^32^P]ATP into Pah1 in a total volume of 20 μl. The reaction mixture contained 50 mM Tris-HCl (pH 7.5), 10 mM MgCl_2_, 2 mM dithiothreitol, 100 μM [γ-^32^P]ATP (∼3000 cpm/pmol), 120 ng Rim11, and 1 μg Pah1. The kinase reactions were terminated by the addition of 6.7 μl 4x Laemmli sample buffer, followed by SDS-PAGE (119) to resolve ^32^P-labeled Rim11 or Pah1 from radioactive ATP. Phosphorylated Rim11 or Pah1 was visualized by phosphorimaging using a Storm 860 Molecular Imager (GE Healthcare), and the extent of phosphorylation was quantified by ImageQuant software. The Pho85-Pho80 kinase reaction with the Rim11 peptides and [γ-^32^P]ATP was terminated by spotting the reaction mixture onto a P81 phosphocellulose paper, followed by phosphoric acid washing of the peptide-bound paper and scintillation counting (63).

### Phosphoamino acid analysis

The ^32^P-labeled Rim11, which was resolved by SDS-PAGE, was transferred to a polyvinylidene difluoride membrane and hydrolyzed with 6 N HCl at 100 °C (120). The acid hydrolyzate was mixed with phosphoamino acid standards and separated by two-dimensional electrophoresis on cellulose TLC plates. Radioactive phosphoamino acids were visualized by phosphorimaging analysis using a Storm 860 Molecular Imager (GE Healthcare). Non-radioactive phosphoamino acid standards were visualized by ninhydrin staining.

### LC-MS/MS analysis of protein phosphorylation

Phosphorylation sites on Rim11 targeted by the Pho85-Pho80 kinase complex were identified by LC-MS/MS at the Center for Integrative Proteomics Research, Rutgers University. Rim11 (0.4 mg) was incubated with Pho85-Pho80 (0.1 mg) for 2 h at 30 °C in the absence or presence of 100 mM ATP. Reaction products were separated by SDS-PAGE (119) on a 10% polyacrylamide gel and visualized by Coomassie Blue staining. Excised gel bands were reduced with 10 mM dithiothreitol for 30 min at 60 °C, followed by alkylation with 20 mM iodoacetamide for 45 min at RT in the dark. Proteins were then digested overnight at 37 °C with 0.2 μg of trypsin, chymotrypsin, or Asp-N.

Peptides were extracted twice with 5% formic acid in 60% acetonitrile and subsequently dried under vacuum prior to LC-MS/MS analysis. Peptides were analyzed by nanoLC-MS/MS using a Vanquish Neo UHPLC system (Thermo Fisher Scientific) coupled to a trapped ion mobility spectrometry time of flight high throughput mass spectrometer (Bruker). Samples were directly loaded onto a PepSep 100 analytical column (75 μm × 25 cm; Bruker) and separated at a flow rate of 300 nl/min using a segmented linear gradient consisting of 2 to 15% solvent B over 10 min, followed by 15 to 32% solvent B over 15 min (solvent A: 0.2% formic acid in water; solvent B: 0.2% formic acid in acetonitrile). Data-independent acquisition parallel accumulation-serial fragmentation (DIA-PASEF) data were acquired over an m/z range of 300 to 1200. Precursor ions were sampled using eight diaPASEF scans, each comprising three quadrupole isolation windows and 24 ion-mobility steps spanning a mobility range of 0.7 to 1.3 V s cm^−2^ (1/K_0_). Variable isolation widths of 36 to 41 Th were applied at each mobility step. The acquisition time for each diaPASEF scan was 85 ms, resulting in a total cycle time of approximately 0.7 s.

Database searches were performed against a target-decoy FASTA database comprising the *S. cerevisiae* UniProt proteome and a database of common laboratory contaminants using FragPipe v22.0 with the label-free quantitative phosphoproteomics workflow. Search parameters included precursor and fragment mass tolerances of ± 20 ppm, carbamidomethylation of cysteine as a fixed modification, and oxidation of methionine together with phosphorylation of serine, threonine, and tyrosine residues as variable modifications. Protease specificity was set to strict trypsin, chymotrypsin, or Asp-N cleavages, allowing up to two missed cleavages. Only phosphorylation sites with a Best Localization probability >0.75 for serine, threonine, tyrosine (+79.9663 Da) modifications were considered for further analysis. For quantification of Rim11 phosphorylation, the summed intensities of all phosphopeptides containing a given phosphorylation site were normalized to the total intensity of the corresponding Rim11 protein. The raw data and database results for the peptide analyses of Rim11 are deposited in the MassIVE repository.

### Lipid labeling and analysis

*S. cerevisiae* cells were labeled to steady-state with [2-^14^C]acetate (121), followed by the extraction of the lipid fraction (122) as described previously (123). The extracted lipids were resolved by one-dimensional TLC on silica gel plates using the solvent system hexane/diethyl either/glacial acetic acid (40:10:1, v/v) (124). The resolved lipids were visualized by phosphorimaging (Storm 860 Molecular Imager, GE Healthcare), followed by the image quantification with ImageQuant software using a standard curve of [2-^14^C]acetate. The identity of radiolabeled lipids was confirmed by comparison with the migration of authentic standards visualized by staining with iodine vapor.

### SDS-PAGE and Western blotting

Standard protocols for SDS-PAGE (119) and Western blotting (125, 126) were followed in this work. Normalized gel loading was based on protein concentration (127), and transfer of proteins resolved in the polyacrylamide gel to the polyvinylidene difluoride membrane was monitored by Ponceau S staining. The protein blots were probed with rabbit anti-Cho1 (0.27 μg/ml), mouse anti-Pgk1 (0.05 μg/ml), and rabbit anti-Pah1 (2 μg/ml) antibodies. The secondary alkaline phosphatase-conjugated goat anti-rabbit IgG antibody and goat anti-mouse IgG antibody were used at dilutions of 1:1500 and 1:3,000, respectively, using enhanced chemifluorescence substrate. Fluorescence signals from the immunoblots were acquired by the Storm 860 Molecular Imager (GE Healthcare) and analyzed by ImageQuant TL software (GE Healthcare).

### Protein determination

Protein concentration was determined by the Coomassie Blue binding assay of Bradford with bovine serum albumin as the standard (127).

### Data analysis

The statistical analysis of data using the *t* test was performed with Microsoft Excel software. The *p* values < 0.05 were taken as a significant difference.

## Data availability

Raw phosphorylation data and database search results for Rim11 are deposited in the MassIVE repository (https://massive.ucsd.edu/ProteoSAFe/static/massive.jsp) with the accession number MSV000101661. All other data are contained within the article.

## Conflict of interest

The authors declare that they have no conflicts of interest with the contents of this article.
